# Unwinding the mechanism of macrophage repolarization potential of *Oceanimonas* sp. *BPMS22*-derived protein protease inhibitor through Toll-like receptor 4 against experimental visceral leishmaniasis

**DOI:** 10.3389/fcimb.2023.1120888

**Published:** 2023-03-22

**Authors:** Adithyan Jayaraman, Sujatha Srinivasan, Kiran Babu Uppuluri, Santanu Kar Mahapatra

**Affiliations:** ^1^ Department of Biotechnology, School of Chemical and Biotechnology, SASTRA Deemed to be University, Thanjavur, India; ^2^ Department of Paramedical and Allied Health Sciences, Midnapore City College, Midnapore, India

**Keywords:** *Oceanimonas* sp. *BPMS22*, immunotherapy, innate immune response, visceral leishmaniasis, macrophage repolarization, Toll-like receptor 4

## Abstract

The *Oceanimonas* sp. *BPMS22*-derived protein protease inhibitor (PPI) has been proven to shift macrophages towards an inflammatory state and reduce *Leishmania donovani* infection *in vitro* and *in vivo*. The current study explored and validated the mechanistic aspects of the PPI and Toll-like receptor (TLR) interaction. The PPI exhibited the upregulation of TLR2, TLR4, and TLR6 during treatment which was proven to orchestrate parasite clearance effectively. An *in silico* study confirmed the high interaction with TLR4 and PPI. Immune blotting confirmed the significant upregulation of TLR4 in macrophages irrespective of *L. donovani* infection. Pharmacological inhibition and immune blot study confirmed the involvement of the PPI in TLR4-mediated phosphorylation of p38 MAPK and dephosphorylation of ERK1/2, repolarizing to pro-inflammatory macrophage state against experimental visceral leishmaniasis. In addition, in TLR4 knockdown condition, PPI treatment failed to diminish M2 phenotypical markers (CD68, Fizz1, Ym1, CD206, and MSR-2) and anti-inflammatory cytokines (IL-4, IL-10, and TGF-β). Simultaneously, the PPI failed to upregulate the M1 phenotypical markers and pro-inflammatory cytokines (IL-1β, IL-6, IL-12, and IFN-γ) (*p* < 0.001) during the TLR4 knockdown condition. In the absence of TLR4, the PPI also failed to reduce the parasite load and T-cell proliferation and impaired the delayed-type hypersensitivity response. The absence of pro-inflammatory cytokines was observed during a co-culture study with PPI-treated macrophages (in the TLR4 knockdown condition) with day 10 T-cell obtained from *L. donovani*-infected mice. This study supports the immunotherapeutic potential of the PPI as it interacted with TLR4 and promoted macrophage repolarization (M2–M1) to restrict the *L. donovani* parasite burden and helps in the mounting immune response against experimental visceral leishmaniasis.

## Graphical Abstract

Graphical representation which illustrates that PPI induces TLR-4, resulting in the phosphorylation of p38 and downregulation of ERK-1/2-associated M2 to M1 polarization towards anti-leishmanial immune responses.

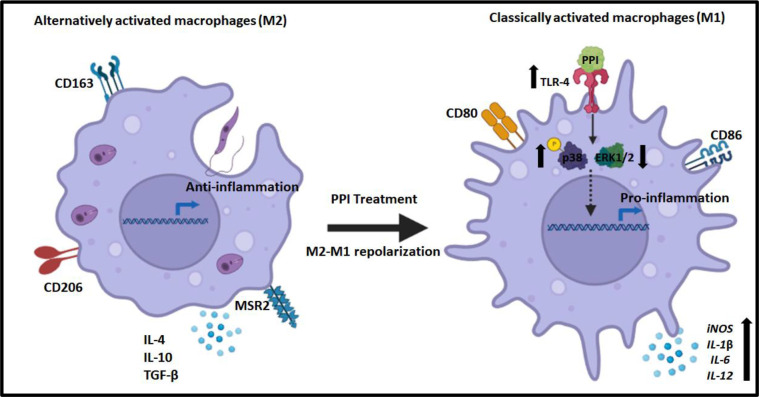

## Highlights

(1) *BPMS22*-derived PPI activates TLR-4-mediated innate immune response against visceral leishmaniasis.
(2) *BPMS22*-derived PPI-mediated phosphorylation of p38 results in the secretion of pro-inflammatory cytokines.
(3) *BPMS22*-derived PPI could be a potent TLR-4 agonist and modulator of the immune response through M2–M1 repolarization in controlling *L. donovani* infection inside macrophages.

## 1 Introduction

Visceral leishmaniasis (VL), also known as “black fever”, is caused by *Leishmania donovani*, an obligate, intracellular protozoan parasite that resides inside the host macrophage for survival. The common symptoms include hepatosplenomegaly, pyrexia, weight loss, hyperglobulinemia, and pancytopenia, which lead to death (Gazzinelli et al., 2004). VL is endemic in tropical and subtropical regions of Brazil, Bangladesh, India, Nepal, and Sudan. The drawbacks of existing drugs are toxicity, high cost, and failure of VL treatment due to the development of resistance against drugs like amphotericin B (Alvar et al., 1997).. Several studies indicate the potential involvement of host innate immune response during *Leishmania* infection through the development of Th1-type response (IFN- γ, IL-6, IL-12, and TNF-α) over Th2-type responses (IL-4, IL-10, TGF-β). The Th1-type response is significant for activating classical or pro-inflammatory macrophages (M1) and nitric oxide production, which is detrimental to the parasite. Th2-type response helps activate alternative or anti-inflammatory macrophages (M2), and the secretion of immune regulatory cytokines helps in the pathogenesis of VL (Mukhopadhyay et al., 2015; Raja et al., 2021).

The determining factor for the two types of immune response is initiated by the host’s innate immune system through Toll-like receptors (TLRs). TLRs are the foremost defensive mechanism against infectious diseases (Gazzinelli et al., 2004). TLRs confer specificity to the host’s innate immune cells by recognizing various components of the pathogen that causes the disease. Broad microbial recognition of TLRs develops through evolution. The TLR family, comprised of 12 members (TLR1–TLR9 and TLR11–TLR13), has specificity for several pathogens and involves the production of cytokines and other effector molecules. In this context, TLRs recognize the *Leishmania* parasite through lipophosphoglycan (LPG), glycoinositolphospholipids (GIPL), and GP63, which results in the early pro-inflammatory to the later anti-inflammatory state of macrophages and supports in parasite residence (Tomiotto-Pellissier et al., 2018). TLRs possess both beneficial and detrimental characteristics based on their activation. Among other TLRs, TLR4 has been shown to be beneficial to the host through pronouncing pro-inflammatory production *via* activating M1 macrophages and Th1 cells, resulting in the clearance of parasites. Studies have shown the importance of TLR4 in parasite clearance by nitric oxide, which is lethal to the *Leishmania* parasite (Gatto et al., 2015). The elastase produced by neutrophils activates TLR4 activation and helps in parasite killing (Dias et al., 2019). Mice with a TLR4 gene mutation fail to heal *Leishmania* cutaneous lesions (Kropf et al., 2004). Moreover, TLR4 activation helps in T-cell activation and clonal expansion, facilitating isotype switching, B cell maturation, and robust immunoglobulin production (Pone et al., 2010). TLR4 agonists extended application as nanotherapeutic agents or vaccine adjuvants. Monophosphoryl lipid A used in vaccine preparation and aminoalkyl glucosaminide phosphates in the clinical trial are successful non-toxic TLR4 agonists compared with lipopolysaccharides (LPS) (Evans et al., 2003; Casella and Mitchell, 2008).

Since there has been a lack of medicine developed in the last 40 years, immunotherapy provides a prospective therapeutic strategy for the treatment of active leishmaniasis. Adjuvant vaccines and immunotherapy for leishmaniasis have shown encouraging effects. The adjuvants appear to boost vaccination treatment and immunotherapy effectiveness by stimulating cellular immunity *via* TLRs (Alderson et al., 2006). Besides leishmaniasis, TLR4 activation during immunotherapy has been linked to better prognosis against several infectious diseases and cancer models (Karmakar et al., 2012; Kaczanowska et al., 2013; Rostamian and Niknam, 2017). TLR activation leads to high levels of IFN-γ and the early production of IL-12 (Das et al., 2013). These suggest the link between TLR and immunotherapy for *Leishmania* infection.

We have recently reported a protein protease inhibitor (PPI) purified from *Oceanimonas BPMS22* sp. that showed better IC_50_ of 25.28 ± 1.675 and 0.415 ± 0.015 μg/ml against both promastigote and amastigote, respectively. The PPI exhibited potent immune modulation shifting from anti-inflammatory to the increased pro-inflammatory status of macrophages (M2–M1) in the pre-stimulated macrophages and *Leishmania* infection model (Jayaraman et al., 2022). In the current study, we have unraveled the underlying mechanism behind the PPI-mediated repolarization of M2–M1 macrophages with the help of TLR-4 siRNA/shRNA. The PPI has been demonstrated to be a potent TLR4 agonist and is also involved in MAPK activation, which reveals its immunotherapeutic potential against VL.

## 2 Materials and methods

### 2.1 Materials

RPMI-1640 medium was procured from HiMedia. The ELISA kits for IFN-γ, IL-2, IL-10, IL-12, TNF-α, and TGF-β were purchased from R&D Systems. Fetal bovine serum (FBS) and 100x Pen-Strep were purchased from Gibco, Griess reagent and oligo primers were from Sigma, cDNA synthesis kit and RT-PCR chemicals were from Thermo, and real-time PCR (SYBR Green) was from TAKARA Bio. TLR4 si-RNA and TLR4 shRNA were procured from Santa Cruz Biotechnology, USA.

### 2.2 Parasite maintenance, cell culture, and animal conditions

*Leishmania donovani* AG83 strain (MHOM/IN/AG/83) was cultured in M199 media supplemented with 10% FBS and 1X pen-strep solution. The virulence of *L. donovani* AG83 was maintained through regular passaging in BALB/c mice. Peritoneal macrophages were isolated from BALB/c mice injected with a thioglycolate broth and cultured in RPMI-1640 with 10% FBS and 1X Pen-Strep antibiotics in 5% CO_2_ incubator (Cell Xpert, Eppendorf) at 37°C. Age, sex, and weight-matched BALB/c mice were purchased from the Central Animal Facility, SASTRA Deemed to be University (NABL accredited). BALB/c mice were used for the experiments with prior approval of the Institutional Biosafety Committee, and the Institutional Animal Ethics Committee (IAEC) (approval number: 671/SASTRA/IAEC/RPP, dated 21/11/2020) approved all animal studies.

### 2.3 Protein–protein interaction of Toll-like receptors of humans and BPMS22–PPI

The constructed 3D model of BPMS22–PPI was proposed to interact with the TLRs of humans using Cluspro v2.0. This server dock involves three steps. First, rigid body docking is performed by sampling billions of confirmations using the fast Fourier transform correlation. Second, the best models with the docking complex are represented by interface root mean square deviation based on the 1,000 lowest energy structures to build the most significant clusters. Third, removing steric conflicts by energy minimization validated the stability of the docking model (clusters). Pymol software was used to visualize the protein–protein interaction of BPMS22-PPI and the TLRs. The BPMS22–PPI interaction residues to TLRs were implemented using the DIMPLOT software from Ligplot version 2.1 (Dey et al., 2019; Kumar et al., 2020).

### 2.4 Generation of TLR4 knockdown macrophages

Peritoneal macrophages were transfected with siRNA for TLR4 (SC-40260) or scrambled siRNA as a control and allowed to transfect using a transfection medium (SC-36868) as per the manufacturer’s instructions (Santa Cruz Biotechnology). The experiments were carried out after 48 h of transfection.

### 2.5 Quantification of the mRNA expression of M1 and M2 signatures, co-stimulatory molecules, TLRs, and cytokines

The PPI-mediated changes in the mRNA expression of *L*. *donovani*-infected macrophages were studied using real-time PCR. After completion of treatment, the macrophages were collected in Trizol, and total RNAs were extracted. A total of 1 μg RNA was used to prepare cDNA using Revert Aid M-MuLV Reverse Transcriptase (Fermentas). cDNA was analyzed by real-time PCR using Takara SYBR green kit. In a 96-well real-time plate (Applied Biosystems), first, a master mix with primers of specific genes followed by SYBR green was added, and finally, cDNA was added to the wells. The PCR reaction was set in a real-time machine (Eppendorf), and C.T. values were taken after the completion of the reactions. Fold change was calculated by 2^-^ **^ΔΔCt^** methods (Kar et al., 2021b). The gene-specific oligos are given in **Supplementary Tables S1**, **S2**.

### 2.6 Anti-amastigote assay

In total, 1 × 10^5^ peritoneal macrophages from mice (BALB/c) were cultured in eight-well-chamber glass slides (Genetix) per 200 μl of RPMI1640 supplemented with 10% FBS. After 24 h of post-seeding, the adhered and resting macrophages were infected with promastigotes at a macrophage/promastigote ratio of 1:10 for 6 h at 37°C with 5% CO_2_ in an incubator (CellXpert, C170 Eppendorf). The cells were washed twice with RPMI1640 media to remove non-internalized promastigotes. The infected macrophages were cultured for another 18 h and treated with PPI (1 μg/ml) for 48 h. The macrophages were washed, fixed in ice-cold methanol, and stained with Giemsa stain. Parasite load was enumerated as the number of amastigotes per 200 macrophages under a light microscope (BX43, Olympus). The percentage of parasite killing was determined as a relative percentage of infected control (Raja et al., 2017).

### 2.7 Nitrite release in macrophages

Nitric oxide generation was estimated in the culture supernatant after treatment of the infected macrophages with the PPI (1 μg/ml). Splenocytes were prepared as per standard protocol and re-stimulated with soluble leishmanial antigen (SLA; 10 µg/ml). Nitrite accumulation was measured calorimetrically using Griess reagent (Sigma). Absorbance was taken at 550 nm using a microplate reader (Biotek, Synergy H1). Nitrite generation was calculated using the standard curve prepared from NaNO_2_ as the analyte, and data were expressed in micromoles of nitrite (Kar et al., 2021a).

### 2.8 Arginase 1 activity in macrophages

Macrophage lysates and splenocytes from *in vivo* sets treated with the PPI (1 μg/ml) were prepared as per standard protocol and re-stimulated with SLA (10 µg/ml). Arginase 1 activity was measured using the arginine hydrolysis method. The urea concentration was measured at 540 nm after adding 40 μl of α-isonitrosopropiophenone (dissolved in 100% ethanol), followed by heating at 95°C for 30 min. One unit of enzyme activity was defined as the amount of enzyme that catalyzed the formation of 1 mol of urea/min (Raja et al., 2021).

### 2.9 Cytokine analysis by sandwich ELISA

The PPI-mediated changes in cytokine releases were estimated from the cell-free supernatant of untreated and treated macrophages. Sandwich ELISA was performed with respective cytokines ELISA kits (R&D system) according to the manufacturer’s instructions.

### 2.10 Generation of TLR4 knockdown mice

Male BALB/c mice (6–8 weeks, weight-matched) were allowed to transfect with TLR4 shRNA or control shRNA intravenously through the lateral tail vein of mice. After 2 days, mice that remained uninfected were infected with 2 × 10^7^ *L. donovani* stationary-phase promastigotes through the lateral tail vein. After 15 days of infection, the PPI (1 mg/kg of B.W.) was administered intravenously *via* the tail vein for three regimens in 3-day intervals. After the completion of treatment, all the mice were sacrificed on the 14th day from the last day of the treatment. Then, hepatic parasite burden was determined from tissue imprints after Giemsa staining. As described previously, results were expressed in Leishman-Donovan units (LDU) (Tang et al., 2018).

### 2.11 T-cell proliferation

Spleens from the different groups of BALB/c mice were collected, homogenized into single-cell suspension, and added to a top-on density gradient histopaque solution in a ratio of 1:1 and centrifuged using a swing bucket rotor for 45 min at 250 g. Splenocytes were collected from the plasma histopaque interface, washed using phosphate-buffered saline (PBS) twice, and seeded in 96-well plates. In total, 5 × 10^5^ T-cells were seeded in 96-well plates in triplicate and stimulated with 10 µg/ml of SLA for 72 h. The T-cell proliferation assay was conducted using resazurin. The resazurin assay was then used to assess proliferation. IL-2 was measured using sandwich ELISA (Raja et al., 2021).

### 2.12 Delayed-type hypersensitivity response

Soluble leishmanial antigen was prepared using stationary-phase *L. donovani* promastigote. The promastigotes were lysed using probe sonication, and the supernatant was collected and used as SLA after the estimation of protein concentrations using the Bradford protein assay method (Bio-Rad, Hercules, CA, USA). The prepared SLA antigen was aliquoted and kept at −80°C for future use. Delayed-type hypersensitivity (DTH) response was determined by measuring the difference in footpad swelling, followed by an intradermal injection in the test footpad with 50 µg of SLA compared with contralateral (PBS-injected) footpad in different groups of animals. After 72 h, net footpad swelling was measured using vernier calipers (Raja et al., 2021). The measurement was compared with the control group.

### 2.13 Macrophage/T-cell co-culture study

Macrophages were seeded in 24-well cell culture plates at 5 × 10^5^ cells per well. After 24 h, the macrophages were infected with *L. donovani* promastigotes in a 10:1 (parasites/macrophages) ratio. The non-adherent parasites were washed out by PBS and treated with PPI at 1 µg/ml for 24 h. Afterward, the macrophages were co-cultured with or without D10T-cell (T-cells were separately isolated after the 10th day of *L. donovani*-infected mice) at 10:1 (T-cells/macrophages) for 48 h. After that, supernatants were collected and used for the Th1 (IFN-γ and IL-12) and Th2 (IL-10) cytokine secretion using ELISA (Ettinger and Wilson, 2008).

### 2.14 Immunoblot analysis

BALB/c-derived peritoneal macrophages were seeded in six-well plates, infected with *L. donovani* stationary-phase promastigotes, and treated with PPI. Then, the adherent cell populations were collected from the plate and centrifuged at 500 g for 15 min at 4°C. The cell pellet was dissolved in RIPA buffer, and the cell suspensions were sonicated using a probe sonicator. The sample was centrifuged at 14,000 *g* for 15 min at 4°C, and the protein in samples was estimated using the Bradford method. Then, 50 μg of the total protein was subjected to 10% SDS PAGE gel electrophoresis and transferred to a polyvinylidene (PVDF) membrane. The PVDF membrane was blocked by 5% milk protein in Tris-buffered saline with 0.1% Tween-20 (TBS-T), and immunoblotting was performed to evaluate the levels of protein expressions. The antibody of TLR-4 (SC-293072), p-p38 (SC-166182), pERK (SC-7383), p38 (SC-535), ERK1/2 (SC-514302), and GAPDH (SC-365062) and a secondary antibody of anti-mouse IgG HRP (SIGMA-A9917) served as an internal control. The immunoreactive band was captured by the Bio-Rad Gel Doc documentation system after being visualized with an ECL kit (Bio-Rad), and band intensities were analyzed using ImageJ software (Raja et al., 2017).

### 2.15 Anti-amastigote assay in the presence of TLR4 siRNA and pharmacological inhibitors

BALB/c-derived peritoneal macrophages were infected and treated with PPI in the presence or absence of p38MAPK inhibitor (SB203580) and iNOS-2 inhibitors (LNMMA). The macrophages were washed, fixed in methanol, and stained with Giemsa stain. Parasite load was calculated as the number of amastigotes per 200 macrophages under a light microscope (BX43, Olympus). The percentage of parasite killing was determined relative to the infected control. NO generation was measured in the presence of TLR 4 siRNA or inhibitors (Kar et al., 2021a).

### 2.16 Statistical analysis

All the *in vitro* experiments were performed in triplicates and repeated two to three times. *In vivo* experiments were conducted with *n* = 6 mice per group. The data were presented as mean values ± standard deviation (SD) as prepared in GraphPad prism 6.0. Statistical analysis was performed by analysis of variance of (ANOVA) (two-way ANOVA) followed by the Tukey test.

## 3 Results

### 3.1 PPI effect on the mRNA expression of TLRs in *L. donovani-*infected macrophages

**Figure 1:**
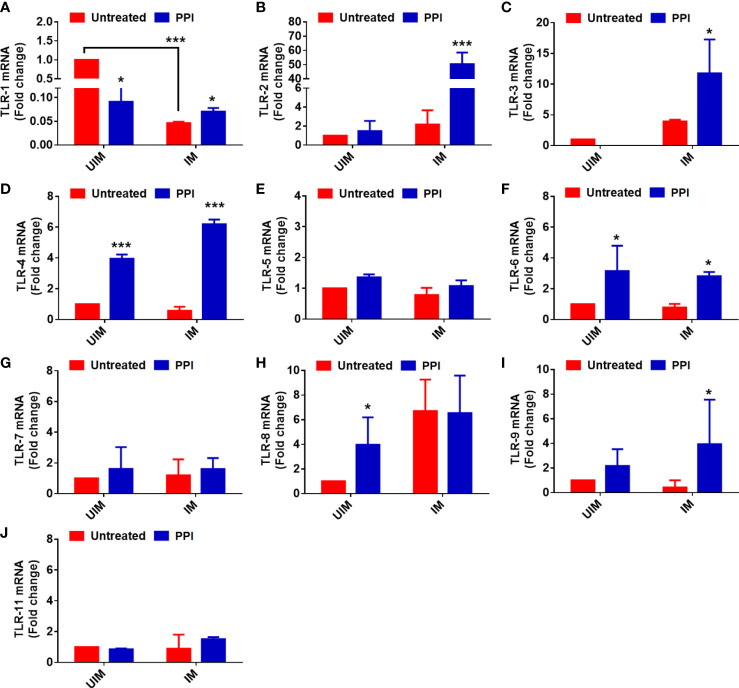
Effect of the protein protease inhibitor (PPI) on the mRNA expression of Toll-like receptors (TLRs) in uninfected macrophages (UIM) and infected macrophages (I.M.). **(A–J)** Macrophages were cultured and were infected with stationary-phase *L. donovani* promastigotes in a 1:10 (macrophage/parasite) ratio. After 4 h, non-ingested parasites were removed by washing with sterile phosphate-buffered saline. After 24 h of infection, the macrophages were treated with or without PPI. After 4 h of PPI treatment, the cells were collected in Trizol. The mRNA expression of TLR-1 to TLR-11 was determined in triplicate using real-time PCR. All the experiments were repeated at least two times, and data from one representative experiment were shown here as mean ± SD of relative fold change compared with uninfected control. The asterisks indicate a significant difference upon comparing the respective untreated PPI and the treated PPI groups. *p ≤ 0.05 and ***p ≤ 0.001.

TLRs are a class of transmembrane receptors that have an important role in the innate immune system and are often the first receptors to recognize the molecular patterns of pathogens (Chauhan et al., 2017). Immune responses initiated by TLRs have a major role in the outcome of leishmaniasis; it can be beneficial and detrimental to the host. Macrophages were allowed to be infected, and with PPI treatment, we studied the mRNA expression of the different TLRs in uninfected and infected macrophages. TLR-4, TLR-6, TLR-8, and TLR-9 were increased in uninfected macrophages, while a significant (*p* < 0.05) induction of TLR-1, TLR2, TLR-3, TLR-4, TLR-6, and TLR9 was observed in *L. donovani*-infected macrophages after treatment with PPI. Among these, only TLR-4 expression was enhanced in higher significance (*p* < 0.001) levels in both uninfected and *L. donovani*-infected macrophages (**Figure 1**). The report indicated that activation of TLR-2, TLR-4, TLR-6, and TLR-9 could exert an anti-leishmanial immune response by the surge of pro-inflammatory cytokines and iNOS2-mediated nitrite generation (Bhattacharya et al., 2010; Mukherjee et al., 2012; Pandey et al., 2014). PPI-induced iNOS2 mRNA expression and nitrite release in macrophages (Jayaraman et al., 2022) and enhanced the TLR-4 expression. We may consider the possibility of TLR4 as the target of the PPI to induce the anti-VL immune response. However, TLR2 expression was seen here to be very high as it might play a dual role in inflammation and anti-inflammation, contributing to the pathogenesis of VL before treatment and the protective immune response after treatment (Gatto et al., 2015).

### 3.2 PPI promoted TLR4-MAPK- NOS2 in *L. donovani*-infected macrophages

**Figure 2:**
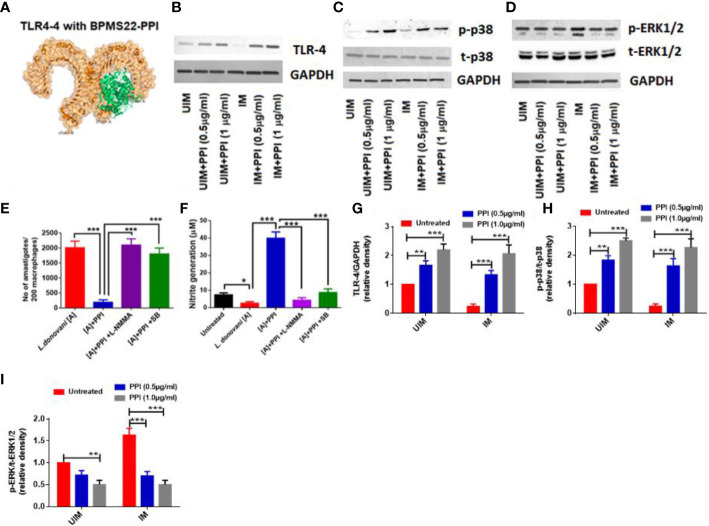
Effect of the PPI on TLR-4, p38MAPK, and ERK1/2 expression in *L. donovani*-infected macrophages. BPMS22–PPI and TLR4 interaction *in silico* **(A)**. BALB/c-derived macrophages were infected with *L. donovani* promastigotes, followed by treatment with the protein protease inhibitor (PPI). After 30 min of treatment, the protein was extracted and subjected to western blotting to study the expression of TLR4, p-p38MAPK, and p-ERK1/2 **(B–D, G–I)**. In a separate set of experiments, macrophages were infected with *L. donovani*. They were pre-treated with a p38 inhibitor (SB203580 at 5 μg/ml) and iNOS inhibitor (L-NMMA at 0.4 mM) for 1 h, followed by the PPI treatment. At 48 h post-treatment, an anti-amastigote study was performed by the Giemsa staining method **(E)**, and NO generation was performed by Griess assay **(F)**. Data are expressed as mean ± SD from triplicate experiments. *p < 0.05, ** *p* < 0.01*** *p* < 0.001.

We have studied the interaction with host receptors of pathogen-associated molecular patterns, *i*.*e*., Toll-like receptors. TLRs are transmembrane glycoproteins that function as a barrier for other molecules entering cells and act as molecular sensors for pathogenesis and immunomodulation. It protects host cells engaged in innate immunity, which paves through adaptive immunity (Chauhan et al., 2017). The docked complex generated a low binding energy for PPI interaction with different TLRs of host origin. The results indicated that BPMS22-derived PPI had the highest interaction with TLR4, with a weighted score of (–)1,344 kcal/mol. The study on TLR mRNA expressions and the *in silico* TLR4-PPI interaction reveal the involvement of TLR-4 in macrophage repolarization after PPI treatment (**Figure 2A**, **S1**). To confirm these results, we have studied the TLR4 expression using Western blotting (**Figure 2B**).

The phosphorylation of p38 in MAPK signaling controls *L. donovani* infection in infected macrophages through nitric oxide and pro-inflammatory cytokines, as proven in our previous work (Raja et al., 2021). From immunoblot analysis, **Figure 2C, G** indicates that *L. donovani* parasite was associated with the decreased phosphorylation of p38 and increased phosphorylation of ERK1/2 (**Figures 2D, I**). However, the PPI enhanced the phosphorylation of p38MAPK in infected macrophages and diminished the phosphorylation of ERK1/2 in a dose-dependent manner. It was previously observed that the PPI increased NO generation through the expression of iNOS2 in peritoneal macrophages. As indicated in **Supplementary Figure S1**, iNOS2 was highly expressed in the PPI macrophages, whereas arginase-1 was overexpressed only in the infected macrophages.

To confirm the involvement of p38MAPK phosphorylation and NOS2 expression in the PPI treatment, we studied *in vitro* anti-amastigote activity and NO generation in macrophages with pharmacological inhibitors. The results confirmed that PPI-mediated parasite killing in infected macrophages was completely revoked in the presence of a MAPK inhibitor (SB203580) and an iNOS inhibitor (L-NMMA) (**Figure 2E**). Simultaneously, nitric oxide production was abolished in the presence of p38 and NO inhibitors in PPI-treated macrophages (**Figure 2F**). In light of the above-mentioned findings, we have confirmed the involvement of p38MAPK-induced NO generation, which helps in parasite killing in *L. donovani*-infected macrophages.

### 3.3 Involvement of TLR4 during BPMS22–PPI modulating M1–M2 markers

**Figure 3:**
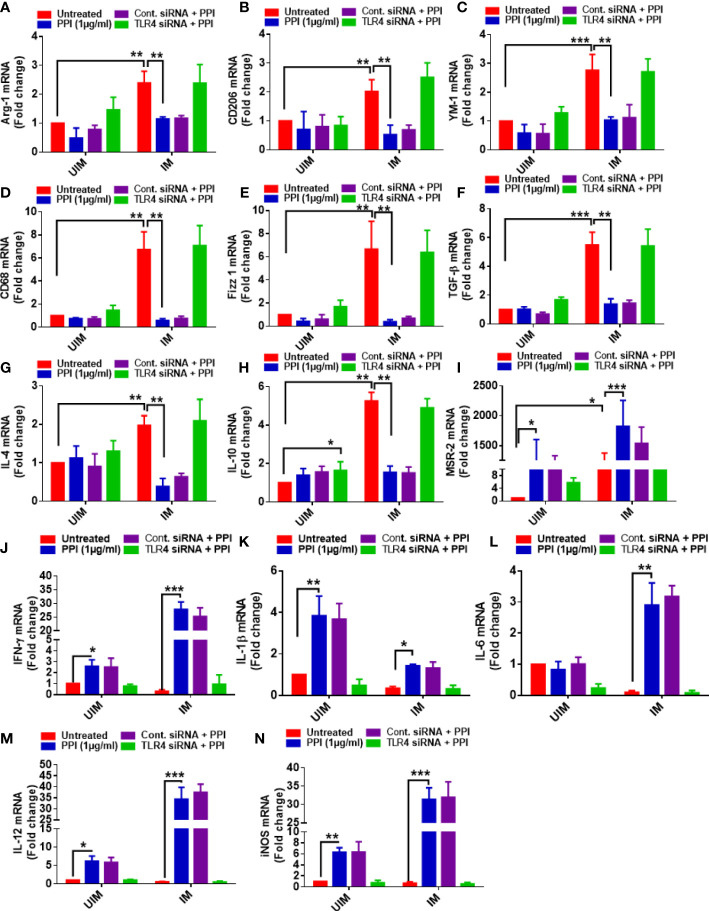
TLR4-dependent alteration of the mRNA expression of M1–M2 markers in *L. donovani*-infected macrophages. The macrophages were cultured and transfected with TLR-4 siRNA. They were infected with stationary-phase *L. donovani* promastigotes in a 1:10 (macrophage/parasite) ratio, and after 4 h, the non-ingested parasites were removed by washing with sterile PBS. After 24 h of infection, macrophages were treated with or without the protein protease inhibitor (PPI). The macrophages were collected in Trizol. The mRNA expression of M1 [arg-1 **(A)**, CD206 **(B)**, YM-1 **(C)**, CD68 **(D)**, Fizz-1 **(E)**, TGF-β **(F)**, IL-4 **(G)**, IL-10 **(H)**, MSR-2 **(I)**] and M2 [IFN-γ **(J)**, IL-1β **(K)**, IL-6 **(L)**, IL-12 **(M)**, iNOS **(N)**] markers was determined in triplicate using real-time PCR. All the experiments were repeated at least two times, and data from one representative experiment were shown here as mean ± SD of relative fold change compared with the uninfected control. The asterisks indicate a significant difference upon comparing the respective PPI-untreated and the PPI-treated groups. *p ≤ 0.05, **p ≤ 0.01, and ***p ≤ 0.001.

Previous experiments found that the BPMS22-derived PPI modulated the M1–M2 markers and shifted the macrophage towards the M1 phenotype (Jayaraman et al., 2022). Moreover, we have also found that the PPI modulated TLR4 in uninfected and infected macrophages over the other TLRs. Hence, we would like to confirm the involvement of TLR4 in PPI-induced M1 polarization and suppression of M2 markers. In these experiments, we have blocked the TLR4 using TLR-4 siRNA. Our results strongly indicated that the PPI failed to enhance the M1 markers (**Figures 3J–N**) and suppress the M2 markers in the presence of TLR4 siRNA (**Figures 3A–I**).

### 3.4 Involvement of TLR4 during BPMS22–PPI modulating Th1–Th2 cytokines

**Figure 4:**
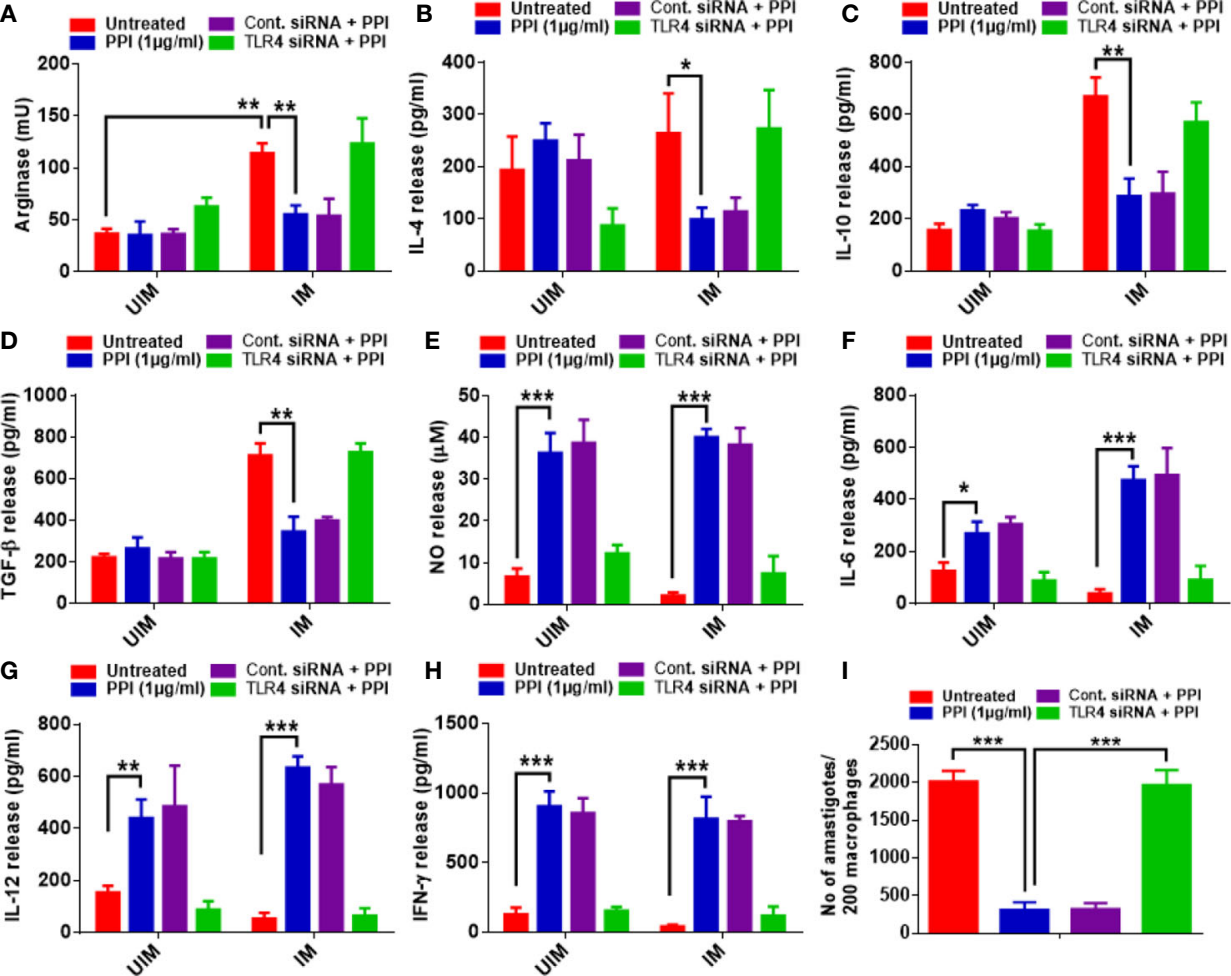
TLR4-dependent alteration of M1–M2 markers and parasite burden in *L. donovani*-infected macrophages. The macrophages were cultured and transfected with TLR-4 siRNA and were infected with stationary-phase *L. donovani* promastigotes in a 1:10 (macrophage/parasite) ratio, and after 4 h, the non-ingested parasites were removed by washing with sterile phosphate-buffered saline. After 24 h of infection, the macrophages were treated with or without the protein protease inhibitor (PPI). The supernatant was subjected to ELISA to determine the level of pro-inflammatory and anti-inflammatory cytokines. Arginase activity **(A)**, IL-4 **(B)**, IL-10 **(C)**, TGF-β **(D)**, NO **(E)**, IL-6 **(F)**, IL-12 **(G)**, IFN-γ **(H)**, Parasite load in macrophage **(I)**. The asterisks indicate a significant difference upon comparing the respective PPI-untreated with the PPI-treated groups. *p ≤ 0.05, **p ≤ 0.01, and ***p ≤ 0.001.

Pro-inflammatory (Th1) and anti-inflammatory cytokines (Th2) play various roles in immune activation and suppression, respectively. Cytokines are responsible for the immune modulation of macrophages during leishmanial infection. An increase in pro-inflammatory production was helpful in parasite clearance, whereas an anti-inflammatory response favors parasite survival. The PPI directed macrophages to the M1 phenotype, which was witnessed in arginase suppression. The PPI failed to diminish arginase expression in TLR4 knockdown macrophages (**Figure 4A**). The PPI suppressed the level of anti-inflammatory cytokines (**Figures 4B–D**) that were withdrawn in the presence of TLR-4 siRNA. The PPI also failed to induce nitric oxide (**Figure 4E**) and pro-inflammatory cytokines in TLR-4-deficient macrophages (**Figures 4F–H**). Interestingly, the effect of the PPI in decreasing the parasite burden in infected macrophages was also abolished (**Figure 4I**). Hence, from this study, we may summarize that TLR4 is the crucial cue for the PPI-induced M1 polarization and decreased parasite load *in vitro.*

### 3.5 BPMS22–PPI cures experimental VL: Involvement of TLR4 *in vivo*

**Figure 5:**
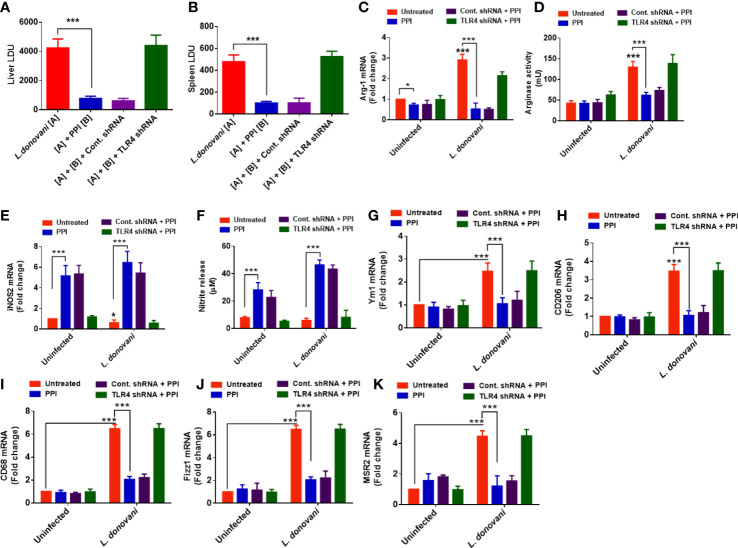
The protein protease inhibitor (PPI) decreased the parasite load in the liver and spleen *via* TLR-4. BALB/c mice (6–8 weeks old) were transfected with TLR4 shRNA, infected with *L. donovani* promastigotes, and treated with the PPI (1 mg/kg B.W.) for a total of four doses in two alternating days. On the 15th day of the last dose of treatment, the mice were sacrificed. Stamp smears were made from liver and spleen tissues and stained with Giemsa, and the parasite load expressed in Leishman Donovan units was enumerated. **(A–B)** The results were expressed as mean ± SD of *n* = 6 for each group. The asterisks indicate a significant difference between the infected control and the PPI-treated group. *p ≤ 0.05, **p ≤ 0.01, and ***p ≤ 0.001. The splenocytes were isolated from each group and re-stimulated with 10 µg/ml of soluble Leishmanial antigen (SLA). **(C, E)** After 48 h of SLA stimulation, the nitrite levels were estimated from cell-free supernatant, and arginase activity was studied from the cell lysates. After 6 h of SLA stimulation, cells were collected in Trizol and the total RNA was isolated. Then, the mRNA expression was studied using real-time PCR with arginase-1 **(C)**, iNOS2 **(E)**, YM-1 **(G)**, CD206 **(H)**, CD68 **(I)**, FIZZ1 **(J)**, and MSR2 **(K)** specific primers. All the results were expressed as mean ± SD. *p < 0.05, and ***p < 0.001.

Furthermore, we have confirmed the involvement of TLR-4 in PPI-mediated anti-VL response *in vivo* in BALB/c mice model. TLR4 shRNA or cont. shRNA were introduced 48 h before *L. donovani* infection in BALB/c mice, followed by treatment with 1.0 mg/kg B.W. of BPMS22–PPI. The *in vivo* dose was selected, as reported earlier (Jayaraman et al., 2022). After 15 days, the mice were sacrificed, and the livers and spleens were analyzed to confirm the stability of TLR-4 knockdown in the TLR-4 shRNA-treated group. The LDU of the liver and spleen parasite load was significantly decreased in the *L. donovani*-infected samples, followed by the PPI-treated group, while in the presence of TLR-4 shRNA, the PPI could not decrease the parasite load in the liver and spleen (**Figures 5A, B**). Aside from that, the PPI also significantly (*p* < 0.01) increased the iNOS-2-dependent NO generation as well as decreased the arginase activity in splenocytes (**Figures 5C–F**). The prompt effector responses remained suppressed in the TLR4 shRNA-treated set, while no changes were found in the cont. shRNA-treated set in the presence of the PPI. Hence, the PPI might abrogate the parasite load in BALB/c mice in a TLR-4 dependent manner. The M2 phenotype markers Ym1, CD206, CD68, Fizz-1, and MSR-2 were affected in the presence of the PPI in *L. donovani*-infected mice, and these were revoked in TLR-4 knockdown mice (**Figures 5G–K**). The results confirm the TLR-4 mediated M2–M1 repolarization by the PPI *in vivo* and restricted liver and spleen parasite burden during experimental VL.

### 3.6 BPMS22–PPI promoted the Th1 response and diminished the Th2 response

**Figure 6:**
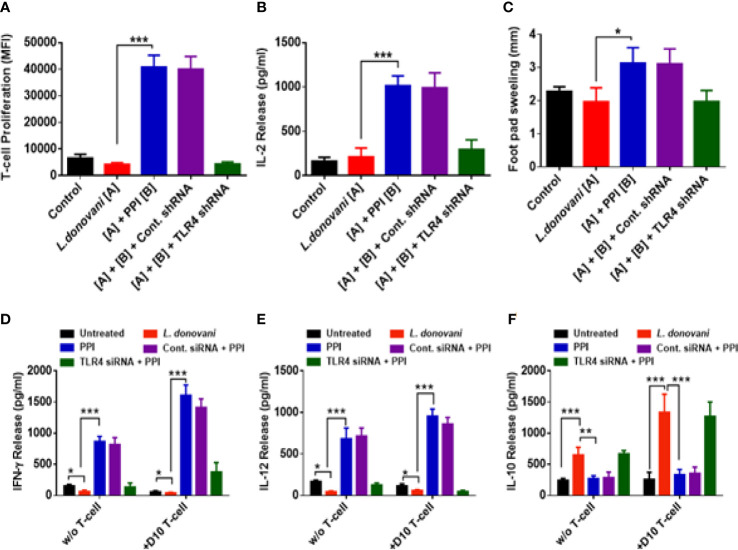
T-cell proliferation and IL-2 release in *L. donovani*-infected BALB/c mice treated with protein protease inhibitor (PPI). BALB/c mice (6–8 weeks old) were transfected with TLR4 shRNA, infected with *L. donovani* promastigotes, and treated with the PPI (1 mg/kg B.W.) for a total of four doses in two alternating days. On the 15th day of the last dose of treatment, the mice were sacrificed. Splenocytes were isolated from each group of mice and re-stimulated with 10 µg/ml of SLA for 72 (h) **(A)** After that, T-cell proliferation was estimated by Alamar blue assay. **(B)** The splenocytes were re-stimulated for 48 h with SLA as before, and the cell-free supernatant was collected, estimating the release of cytokines IL-2 using sandwich ELISA. **(C)** The PPI increased the DTH response in BALB/c mice. **(D)** IFN-γ and **(E)** IL-12 release were increased, and **(F)** IL-10 release was decreased in the co-culture study. The results were expressed as mean ± SD of *n* = 6 animals per group. *p < 0.05, ***p < 0.001.

IL-2 influenced the effector T-cell differentiation and Th1 proliferation which were critical to producing IFN-γ (Bhattacharya et al., 2010). The PPI significantly (*p* < 0.001) enhanced T-cell proliferation and IL-2 release in infected BALB/c mice, which were also suppressed in the presence of the TLR-4 shRNA treatment group (**Figures 6A, B**). DTH indicates the cell-mediated immune response. During a leishmanial infection, macrophage activation is induced by the DTH response, which is important for the host’s defense against the parasite (Wadhone et al., 2009). The PPI significantly (*p* < 0.05) increased the DTH response compared with the untreated infected control (**Figure 6C**). Hence, the PPI could activate the cell-mediated immune response in *L. donovani*-infected BALB/c mice. Interestingly, in the TLR-4 shRNA treatment condition, the DTH responses were absent, confirming the involvement of TLR4 in the PPI-mediated anti-VL response. The PPI increased the Th1 cytokine (IFN-γ, IL-12, and TNF-α) generation significantly (*p* < 0.001) in splenocytes, which were responsible for parasite clearance (**Supplementary Figures S2A–F**). In addition, the PPI significantly (*p* < 0.01) depleted Th2 cytokine IL-10 and TGF-β production in protein and mRNA levels (**Supplementary Figures S2G–J**). Interestingly, the PPI-induced Th1 responses were withdrawn in the TLR-4 shRNA-treated set and failed to diminish Th2 cytokines, confirming the role of the PPI in TLR-4 mediated macrophage repolarization. Th1 cells have a major role in the host cell defense mechanism, and Th2 cells are important for the progression of the parasite burden in the host (Wadhone et al., 2009). The PPI increased the Th1 response (IFN-γ and IL-12) and suppressed the Th2 response (IL-10) (*p* < 0.001) in a co-culture study with D10 T-cells. Hence, PPI-mediated TLR-4 activation involves host protection and is efficient against VL (**Figures 6D–F**). These favored Th1 responses were not observed in TLR4-suppressed conditions.

## 4 Discussion

Despite advancements in critical care treatment, leishmanial infection remains a substantial concern, exacerbated by the threat of antibiotic resistance and the toxicity of drugs. Immunocompromised individuals are more vulnerable to developing a severe infection with *L. donovani*, which frequently leads to the potentially fatal disease of VL (Raman et al., 2010). As a result, immunotherapies targeted at enhancing host immune defenses are extremely appealing options for preventing infection and for the well-being of patients. Recently, there has been mounting evidence that the activation of the innate immune system can confer long-term functional reprogramming in which innate macrophages mount robust responses upon secondary pathogen exposure for more efficient clearance and host protection, a phenomenon known as trained immunity. TLR agonists have been proven to engage in immune modulation *via* modifying signaling cascades, metabolic reprogramming, and epigenetic changes that significantly enhance cell-mediated immune responses towards anti-VL response. Immunomodulatory TLR agonists are also effective adjuvants in vaccination (Akbari et al., 2021). In this study, we evaluated BPMS22–PPI to act *via* TLR-mediated pro-inflammatory production and efficiently combat Leishmanial pathogens *in vitro* and *in vivo*.

In a previous study, BPMS22–PPI was well-proven to shift macrophages toward the pro-inflammation state in the pre-stimulated macrophage and leishmanial infection model (Jayaraman et al., 2022). The expression of iNOS-mediated nitric oxide aids in lowering parasite load in *in vitro* and *in vivo* conditions. Following the previous finding, PPI upregulates TLR 4, 6, 8, and 9 in resting macrophages and TLR 1, 2, 3, 4, 6, and 9 in leishmania-infected macrophages (**Figures 1A–J**). Among other TLRs, cell surface TLRs like TLR 1, 2, 4, 5, and 6 can recognize the broad spectrum of peptides or proteins for their activation, and others are restricted to DNA or RNA as ligands. The upregulation of TLR 2, 4, and 6 expressions was found to be beneficial to the host and detrimental to leishmanial infection. Several reports have shown that the blockade of TLR2 and TLR4 attenuates inflammation in various forms of leishmaniasis (Gatto et al., 2015). At the same time, the long-term activation of TLRs results in uncontrolled inflammation and outcomes in early apoptosis of macrophages. The longevity of the TLR-activated immune response is still unclear. By *in silico* modeling, a possible interaction with PPI was found for TLR-2, TLR-4, and TLR-6. Among others, TLR-4 was found to have a high binding energy with a PPI of (-)1,344 kcal/mol (**Figure 2A**; **Supplementary Figure S1**). Furthermore, protein expression confirms the increase of TLR-4 expression in a dose-dependent manner (**Figure 2A**). In addition, leishmania LPG upregulates the TLR2 expression of macrophages for the persistence of an infection. Multiple TLR expressions would be the consequence of the leishmanial components corresponding to it.

TLR-4 activation leads to the mitogen-activated protein kinase (MAPK) pathway, which consists of proline-directed threonine/serine kinases controlled by upstream phosphorylation cascades. PPI mediates the MAPK pathway as confirmed by failing to generate nitric oxide and reduce parasite load in the presence of a MAPK inhibitor (SB203580) and an iNOS inhibitor (L-NMMA) (**Figures 2E, F**) (Raja et al., 2017; Kar et al., 2021a). Changes in the MAPK signaling pathway, triggered by external and internal stimuli, eventually activate several transcription factors, boosting the transcription of associated genes responsible for cytokines. MAPKs ERK1/2, JNK, and p38 may all play important roles in the production of pro-inflammatory cytokines. The immune blot result implies that the PPI initiates phosphorylation of the MAPK signaling pathways of p38 and downregulates ERK1/2 protein kinases to boost pro-inflammatory cytokines (**Figures 2C, D**) (Shweash et al., 2011).

To confirm the role of the PPI in TLR-4 mediated macrophage activation towards anti-VL immunity, we have accessed the effector function in TLR-4 knockdown macrophages. Nitric oxide is an inflammatory mediator produced by inducible nitric oxide synthase, which can activate macrophages and kill intracellular parasites (Kar et al., 2021a; Raja et al., 2021). Several reports support that TLR-4 is essential for nitric oxide-mediated parasite clearance (Gatto et al., 2015; Carneiro et al., 2021). In contrast, arginase-1 is anti-inflammatory in macrophages and helps parasite survival (Boitz et al., 2017). The association of TLR-4 and arginase-1 is not well studied, but arginase is a reciprocal regulator of nitric oxide synthase involved in nitric oxide production. Interestingly, PPI treatment increased nitric oxide, suppressed arginase-1 expression, and reduced parasite load *in vitro* and *in vivo* in TLR-4 competent macrophages, and the effects were withdrawn in TLR-4 knockdown mice (**Figures 4A, E, I**, **5C–F**).

M1 and M2 phenotype markers were modulated in the presence of the PPI treatment. CD206, Fizz1, Ym1, and CD68 are the hallmarks of M2 macrophage expression markers, and their particular roles in M2 macrophages are poorly understood (Allman et al., 2015; Mukhopadhyay et al., 2015; Tariq et al., 2017). The expression of these markers was diminished in the PPI-treated macrophages, and expressions were revoked in TLR-4 knockdown condition. The exact mechanism of TLR-4-associated M2 marker suppression is not well documented. The PPI directly or indirectly modulates M2 marker expression in macrophages (**Figures 3A–H**).

Cytokines play an essential role in mounting an immune response, thus activating macrophages and recruiting immune cells to the environment. Pro-inflammatory and anti-inflammatory cytokines are responsible for parasite clearance and persistence, respectively. A balance in Th1 and Th2 cytokine response decides the outcome of a leishmanial infection. IFN-γ activates macrophages and monocytes to release oxygen radicals and the secretion of TNF-α, IL-lβ, and IL-6. TNF-α induces granuloma response, and IL-12 restores IFN-γ production and cytotoxic responses in VL. Th1 cytokines are known for host protection against leishmaniasis (Wang et al., 2000; Ben-Sasson et al., 2009; Saha and Silvestre, 2021). Pro-inflammatory cytokines like IFN-γ, IL-1, IL-6, and IL-12 were highly expressed in the presence of the PPI in *L. donovani*-infected macrophages. The effects were withdrawn in the absence of TLR-4 both *in vitro* and *in vivo* (**Supplementary Figures S2A–J**). The PPI mediated an amplified pro-inflammatory cytokine response during a leishmanial infection than un-infected macrophages due to the presence of LPG, GIPL, and GP63 of the leishmanial parasite, in addition to the PPI; these all extend the TLR-4 activation. The T-cell from *L. donovani*-infected mice, on the 10th day, initiates the secretion of pro-inflammatory cytokines through TLR-4 activation when co-cultured with macrophages. The above-mentioned study confirms the PPI’s role in TLR-4-mediated Th1 polarization (**Figures 6D–F**). Several reports support TLR-4-associated pro-inflammatory cytokine secretion (Kropf et al., 2004; Babiker et al., 2015). Anti-inflammatory response by *L. donovani* infection results in the secretion of IL-10, and TGF-β aids in disease progression and immunosuppression through the recruitment of T_reg_ cells (**Supplementary Figures S2G–J**) (Fiorentino et al., 1991; Oswald et al., 1992; Moulik et al., 2021). The PPI could not diminish an anti-inflammatory response in the absence of TLR-4 expression. T-cell proliferation, IL-2 secretions, and DTH response were limited in TLR-4 knockdown mice in the presence of PPI (**Figures 6A–C**). Summarily, the PPI exhibits TLR-4 mediated macrophage repolarization (M2–M1) through MAPKs of the p38 signaling pathway, resulting in the expression of pro-inflammatory cytokines and effectively mounting an immune response against experimental VL.

## 5 Conclusion

TLR4 has been implicated as a critical component in the inflammatory process in response to leishmanial infections since its discovery. TLR4 activation leads to illness resolution in numerous clinical circumstances; nevertheless, when TLR4 activation pathways are poorly controlled, it can contribute to disease progression. More research is needed to determine the role of TLR4 activation in the various stages of leishmanial infections, focusing on the effects of TLR4 signaling on the fine phenotypic changes in macrophages of the innate immune system. In this context, TLR4 targeting by a BPMS22-derived PPI might be an effective method of manipulating macrophages, and the development of molecules acting on TLR4 could represent new disease-modifying therapeutic agents for the treatment of leishmanial infection.

## Data availability statement

The original contributions presented in the study are included in the article/**Supplementary Material**. Further inquiries can be directed to the corresponding authors.

## Ethics statement

The animal study was reviewed and approved by the Institutional Biosafety Committee and Institutional Animal Ethics Committee, SASTRA Deemed to be University (approval number: 671/SASTRA/IAEC/RPP, dated 21/11/2020).

## Author contributions

AJ: methodology, validation, investigation, formal analysis, data curation, writing—original draft, and revision. SS: methodology, validation, investigation, and formal analysis. KU: methodology, formal analysis, data curation resources, writing—review, and editing. SKM: conceptualization, methodology, formal analysis, resources, writing—original draft and revision, writing—review and editing, visualization, supervision, project administration, and funding acquisition. All authors contributed to the article and approved the submitted version.
