# Supplementary material for: Unwinding the mechanism of macrophage repolarization potential of Oceanimonas sp. BPMS22-derived protein protease inhibitor through Toll-like receptor 4 against experimental visceral leishmaniasis
Source: Front Cell Infect Microbiol. 2023 Mar 22;13:1120888. doi: 10.3389/fcimb.2023.1120888 (PMC10073655; doi:10.3389/fcimb.2023.1120888)
Supplement: Supplementary file 1 [file DataSheet_1.pdf]

Supplementary Data/Information

Unwinding the mechanism of macrophage repolarization potential of *Oceanimonas sp. BPMS22*-derived protein protease inhibitor through toll-like receptor 4 against experimental visceral leishmaniasis

Adithyan Jayaraman[1], Sujatha Srinivasan[1], Kiran Babu Uppuluri[1]*, Santanu Kar Mahapatra[1,2]*

[1]Department of Biotechnology, School of Chemical and Biotechnology, SASTRA Deemed to be University, Thanjavur 613 401, India.

[2]Department of Paramedical and Allied Health Sciences, Midnapore City College, Midnapore-721 129, India.

Address for correspondence:

Dr. Santanu Kar Mahapatra: Email: sailar.santanu@gmail.com

Dr. Kiran Babu Uppuluri: kinnubio@gmail.com

Supplementary table S1: TLR Genes, corresponding primer sequences and melting temperature

PCR conditions:

Initial Denaturation: 95°C for 5 min

Denaturation: 95°C for 30 sec

Annealing: @Tm for 30sec

Extension:72°C for 1 min

Total: 40 cycles

Genes	Sequence (5'-3')	Melting temperature (T_m)
TLR-1	F:5'- CAA TGT GGA AAC AAC GTG GA-3'<br>R:5'-TGT AAC TTT GGG GGA AGC TG-3'	60.0°C
TLR-2	F:5'- AAG AGG AAG CCC AAG AAA GC-3'<br>R:5'-CGA TGG AAT CGA TGA TGT TG-3'	62.0°C
TLR-3	F:5'- TCC TTG CGT TGC GAA GTG AA-3'<br>R:5'-TTG GGC GTT GTT CAA GAG GA-3'	66.0°C
TLR-4	F:5'-ACC TGG CTG GTT TAC ACG TC-3'<br>R:5'-CTG CCA GAG ACA TTG CAG AA-3'	52.0°C
TLR-5	F:5'-CAA CCA AAC CAA CGT CAC CCT GTT-3'<br>R:5'-TGG CTT CCT CTT CAT CGC AGT CTT-3'	68.0°C
TLR-6	F:5'- TTG TTT GCG CCC TGG CCT TAA T-3'<br>R:5'-TGT TCT TGG TGG CAG GTC TTT G-3'	67.0°C
TLR-7	F:5'- TGC AAC TGT GAT GCT GTG TGG T-3'<br>R:5'-TTT GAC CTT TGT GTG CTC CTG G-3'	67.0°C
TLR-8	F:5'-TGC CAA ACA ACA GCA CCC AA-3'<br>R:5'-TCA AAG ACT CAG GCA ACC CA-3'	64.0°C
TLR-9	F:5'- ACT GAG CAC CCC TGC TTC TA-3'<br>R:5'-AGA TTA GTC AGC GGC AGG AA-3'	62.0°C
TLR-11	F:5'- CTG AAG GTG AAG GCT TGA GGT T-3'<br>R:5'-CCT GTC ATT TCC AAT CCC AAA-3'	60.0°C
TLR-12	F:5'- TTG AGC TGC AGG GCT TGA TTG T-3'<br>R:5'-GCT TGG TTA GGC CAG TGC TTG AAA-3'	63.0°C
TLR-13	F:5'- ACA CAG TGA ATG GTG CCG ACT T-3'<br>R:5'-AAA GTG GCT GCT GGT GAA CAC T-3'	62.0°C
GAPDH	F:5'-GAG CCA AAC GGG TCA TCA TC-3'<br>R:5'-CCT GCT TCA CCA CCT TCT TG-3'	59.0°C

Supplementary table S2: M1/M2 and cytokines genes, corresponding primer sequences

Genes	Sequence (5'-3')	Melting temperature (T_m)
FIZZ1	F: TGC TGG GAT GAC TGC TAC TG<br>R: CTG GGT TCT CCA CCT CTT CA	55.0°C
MSR-2	F: AAA GTC AGC GTT CCC AGA GA<br>R: GGT GAT GTT CAC GAT TGC AC	53.0°C
CD68	F: GAA AGG GCT TCC CATTTCTC<br>R: TCA AGC CAT TGG TTT TCA CA	51.0°C
YM-1	F: GAA GGA GCC ACT GAG GTC TG<br>R: CAC GGC ACC TCC TAA ATT GT	55.0°C
CD86	F: GTC TGG AGA ATG CTG TGC AA<br>R: GCT CAG ACC TGC CAA AGT TC	54.0°C
CCL-19	F: ATT CCA GTC ACT CCC CTG TG<br>R: GCC TTT GTT CTT GGC AGA AG	53.0°C
CD163	F: CAT GTC TCT GAG GCT GAC CA<br>R: TGC ACA CGA TCT ACC CAC AT	55.0°C
CD206/ MRC1	F: ATG CCA AGT GGG AAA ATC TG<br>R: TGT AGC AGT GGC CTG CAT AG	54.0°C
ARG-1	F: CAG AAG AAT GGA AGA GTC AG<br>R: CAG ATA TGC AGG GAG TCA CC	52.0°C
iNOS2	F: CAG AGG ACC CAG AGA CAA GC<br>R: AAG ACC AGA GGC AGC ACA TC	61.0°C
IL-12	F: CAC GCC TGA AGA AGA TGA CA<br>R: GAC AGA GAC GCC ATT CCA CA	60.0°C
IFN-γ	F: CCT GCT TCA CCA CCT TCT TG<br>R: CGA CTC CTT TTC CGC TTC CT	59.0°C
IL-10	F: TCA CTC TTC ACC TGC TCC AC<br>R: CTA TGC TGC CTG CTC TTA CTC	55.0°C
TGF-β	F: TTG CTT CAG CTC CAC AGA GA<br>R: TGG TTG TAG AGG GCA AGG AC	56.0°C
GAPDH	F: GAG CCA AAC GGG TCA TCA TC<br>R: CCT GCT TCA CCA CCT TCT TG	59.0°C

Supplementary Figure S1: Molecular interaction of PPI and TLRs of host immune system

A.

S.no	Toll-like receptor	Representative	Binding energy
1	TLR-4	Lowest energy	-1344 kcal/mole

B.

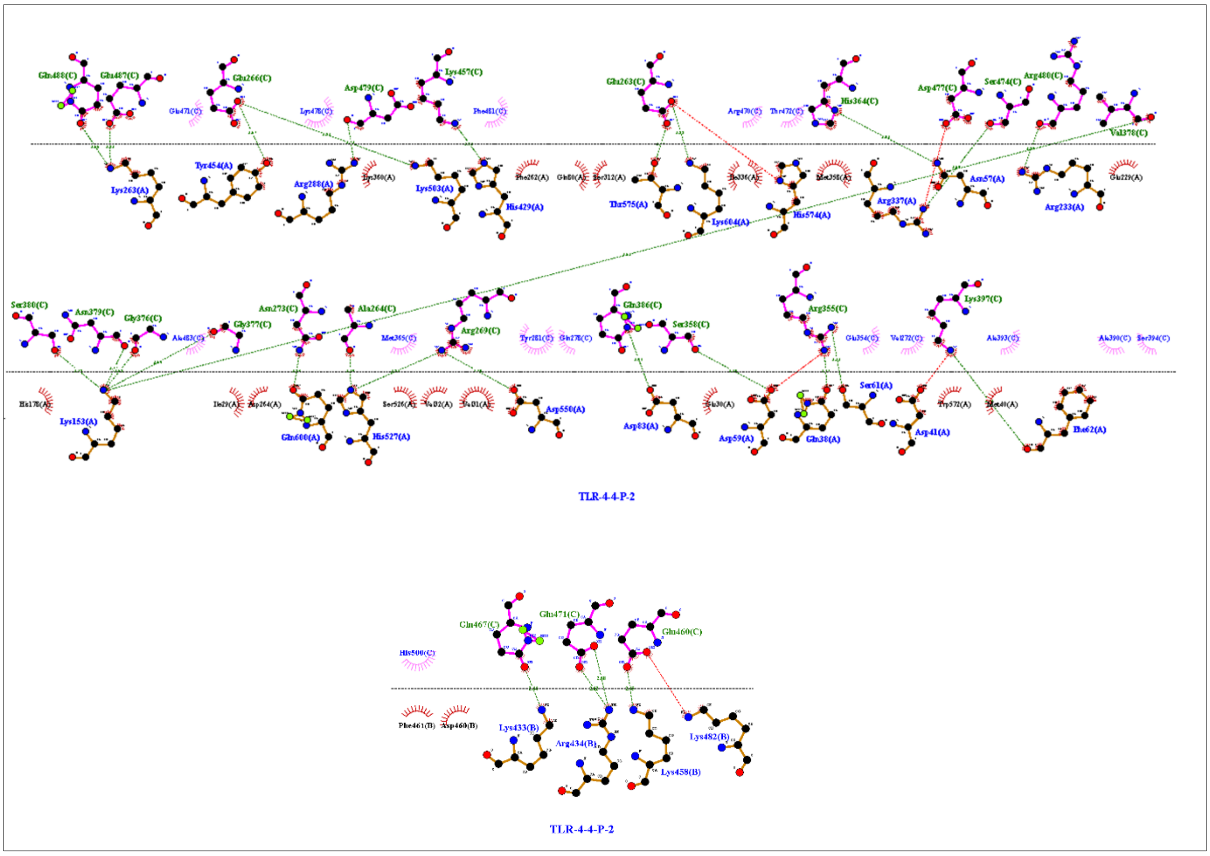

Supplementary Figure S1: Molecular interaction of PPI and TLRs of host immune system using Cluspro v2.0 (A) Table shows binding energy values. The BPMS22-PPI interaction residues to TLRs were implemented using the DIMPLOT software from Ligplot version 2.1. (B) TLR-4-4-BPMS22-PPI

Supplementary Figure S2: Effect of PPI on cytokine secretion in presence or absence of TLR-4 shRNA with or without *L. donovani* infected BALB/c mice model

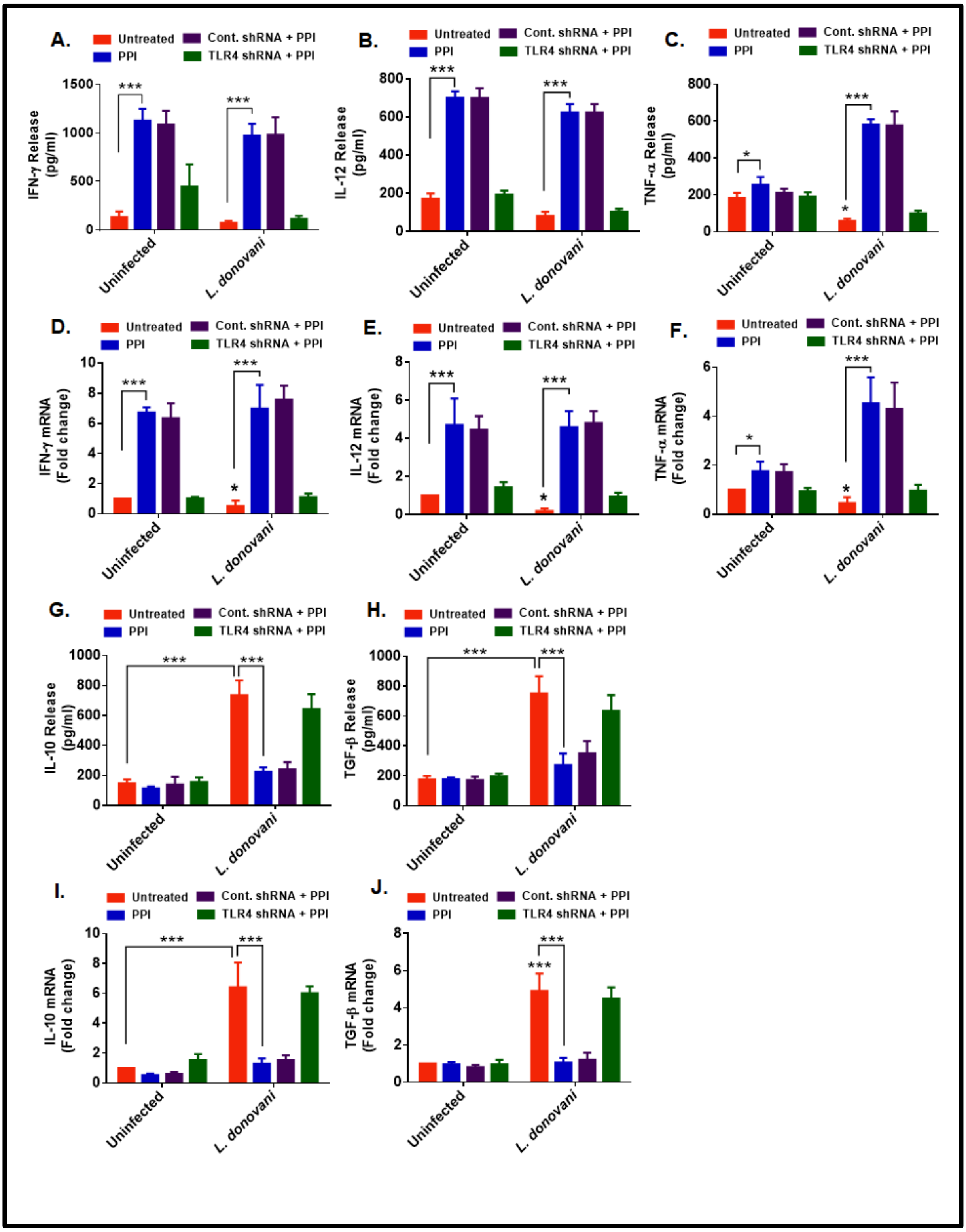

Supplementary Figure S2: Effect of PPI on Th1 and Th2 cytokine response in *L. donovani* infected BALB/c mice. BALB/c mice (6-8 weeks old) were transfected with TLR4 shRNA, infected with *L. donovani* promastigotes, and treated with PPI (1 mg/kg B.W.) for a total of 4

doses in alternative two days. On the 15th day of the last dose of treatment, mice were sacrificed. Splenocytes were isolated from each group and re-stimulated with 10 µg/ml of SLA. After 48 h of SLA stimulation, the level of nitrite and cytokines was estimated from the cell-free supernatant. After 4 h of SLA stimulation, cells were collected in Trizol and isolated the total RNA. Then the mRNA expression was studied by real-time PCR using gene-specific primers. All the results were expressed as mean ± SD. *$p < 0.05$, **$p < 0.01$, and ***$p < 0.001$.
